# Medical History from the Earliest Times

**Published:** 1892-07-09

**Authors:** E. T. Withington


### Medical History from the Earliest Times.

By E. T. Withington, M.A., M.B. Oxon.

### XVI.—The Medical Profession in Ancient Greece.

Physicians in Greece, though classed among the artisans, held from first to last, from the days of Homer to the fall of Constantinople, an honourable position in society. Even Plato, the despiser of medicine, introduces a physician into the highly aristocratic assembly of his "Symposium," and Herodotus attributes to their influence two of the most important events of antiquity, the conquest of Egypt by Cambyses, and the invasion of Greece by the Persians. We have already seen how medical practitioners were valued in Homeric times; in the story of Democedes, we found cities competing for their services and outbidding one another in liberality, and similar evidence is derived at a later period from the decrees in honour of those who have deserved well of the State, a notable proportion of which mention physicians. Thus an inscription, dating from the beginning of the fourth century B.C., found on the Athenian Acropolis, declares that "since Evenor the physician had aforetime shown goodwill to the city and people, and had made himself useful by his art, healing many, both citizens and strangers, and now, having been chosen inspector of drugs, had spent a talent (£240) in that office, it seemed good to the Demos to praise Evenor, son of Evepios the Argive, and to crown him with a crown of green olive for his goodwill to the people of Athens, and that he be an Athenian and his descendants, and that it be lawful for him to enter his name on the list of whatever tribe he pleases, and that this decree be engraved on stone and preserved on the Acropolis." Another inscription of somewhat later date found at Carpathos tells us that Menocritus the Samian had been public medical officer at Brykos more than twenty years, during which period he had been distinguished for zeal and devotion in tending the sick, and had borne himself blameless both in public and private. On the occasion of an epidemic he had rendered special services, had never hesitated to attend patients even outside the city, and had refused payments to which he was justly entitled. Wherefore, "that the people of Brykos may show themselves grateful, and the honourers of good physicians," they decree that Menocritus is worthy of praise, that he shall have a gold crown, and a seat of honour at all their festivals, and that this decree shall be read publicly at the games of Æsculapius, and be engraved on a pillar in the temple of Poseidon. In later times such records become more common but less valuable, for a physician so fortunate as to cure an Antiochus or a Ptolemy was sure to have the freedom of numerous cities laid at his feet. These honours are sometimes mentioned on their tombstones, of which a considerable number have survived, some describing the physician's virtue and skill, others the grief of his relatives and friends, while some show a tendency to that scepticism which has always been attributed to the profession. Thus the physician Nicomedes chose as his epitaph, "I was not, and I became; I am not, and I sorrow not."

In ancient Greece anyone might practise medicine who thought himself qualified to do so and could find patients, but to get a State appointment, he must not only have become distinguished in his profession, but, in Athens at any rate, had to give an account of his teachers and course of education before the Assembly. Practically, every physician began as an apprentice, often to his own father, for medicine, perhaps more than any other, has always been an inherited profession. Celebrated practitioners, however, would naturally attract numerous followers, and thus "schools" arose in various places—Croton, Cyrene, Rhodes, Cos, and Cnidus, till all were superseded by Alexandria. Aristotle distinguishes three classes of physicians—ordinary practitioners, teachers of the art, and amateurs, which last were probably very numerous, for the cultured Greek prided himself upon taking all knowledge as his province.

Having finished his education, the young doctor went on his travels, and settled down in any city which seemed to offer a favourable opening; nor was he ignorant of the plan of working up a practice by treating the poor gratis. Some further attempted to advertise themselves by exaggerating the Hippocratic rule as to elegance of dress and suavity of manner, thereby incurring the ridicule of the satirists, and we hear of no less than four Athenian comedies called "The Doctor," though, unfortunately, little more than their titles have survived. The prosperous physician trained his slaves to act as assistants, and to treat patients of their own class, which they did, according to Plato, in a very rough and ready manner. But slaves might also doctor freemen, for Diogenes tells his master that he ought to obey him though a slave, since even freemen obey slaves when they are physicians or pilots, and a philosopher is better than either. These slaves sometimes purchased liberty by their earnings, and we have an inscription in which such an one agrees to continue as his former master's assistant for a period of five years.

In the Protagoras Plato incidentally tells us how the physician began to examine his patient; "he looks at his face and the tips of his fingers, and then he says, 'uncover your chest and back to me that I may have a better view.'" According to Xenophon, diligent practitioners visited their patients morning and evening, and Galen says that, when at Rome, he went twice a day into the country to see a case of ophthalmia, from which historians have concluded that his practice must have been a very poor one. With regard to fees, classic writers, unfortunately, only give extraordinary cases, such as Pliny's story of the £24,000, which Ptolemy is supposed to have paid Erasistratus or Cleombrotus, but scattered passages in the comic poets seem to indicate that the usual fee for advice and medicine varied from one to two drachmæ (nine to eighteenpence). Sometimes a patient remembered his physician in his will, as did the philosopher Lykon, who bids his heirs "satisfy" his two medical attendants, "who deserve it and more for their zeal and ability." Hippocrates, as we have seen, urged his pupils not to be over-eager for gain, but this warning was naturally often disregarded, and we hear of physicians who never opened their mouths without requiring to be paid for it, and whose first dealing with their patients was always to arrange about the fee. The beautiful Aspasia, of Phocæa, when a girl was horrified by the appearance of a tumour under her chin. A physician was sent for, but, though the girl's parents were poor, he refused to undertake the case till he was paid three staters (about forty-five shillings) on account.

Next in importance to the physician is his office, the Iatreion, which was at once surgery, dispensary, and consulting-room, and which in later times became, like the gymnasia and barbers' shops, a favourite lounging place for idlers. Here the physician gave advice, performed operations, compounded medicines, and sometimes even received resident patients. Every large city also had its public Iatreion, which, as we learn from an inscription found at Delphi, was in some cases supported by a special tax. We have no evidence as to whether these institutions received in-patents, but it would certainly have required very little to convert the free dispensary into a rate-supported hospital.

